# One Health Monitoring of Resistant *Pseudomonas aeruginosa* in Aquatic, Poultry, and Human Sources: Virulence Traits and *bla*_SHV_ Gene Tracking

**DOI:** 10.3390/pathogens14100983

**Published:** 2025-09-28

**Authors:** Ali Wahdan, Mahmoud Ezzat, Amal Emam, Walaa A. Husseiny, Mohamed Abou El-Atta, Ehab M. Abd-Allah, Ahmed M. A. Meligy, Sherief M. Abdel-Raheem, Faisal Almathen, Salah Al-Shami, Saad I. Al-Sultan, Ahmed Alfifi, Wael El-Deeb, Marwa E. Abo Hashem

**Affiliations:** 1 Department of Bacteriology, Immunology, and Mycology, Faculty of Veterinary Medicine, Suez Canal University, Ismailia 41522, Egypt; mezzat05@yahoo.com (M.E.); marwashassan@vet.suez.edu.eg (M.E.A.H.); 2 Veterinary Medicine Directorate, Ministry of Agriculture, Ismailia 41522, Egypt; amalemam222@gmail.com; 3 Department of Animal Wealth Development, Faculty of Veterinary Medicine, Suez Canal University, Ismailia 41522, Egypt; walaa.ali@vet.suez.edu.eg; 4 Central Lab for Aquaculture Research, Abbassa 44662, Egypt; drmohamed.abouelatta@yahoo.com; 5 Veterinary Hospital, Faculty of Veterinary Medicine, Zagazig University, Zagazig 44511, Egypt; zzarad@yahoo.com; 6 Department of Clinical Science, Central Lab, College of Veterinary Medicine, King Faisal University, P.O. Box 400, Al-Hofuf 31982, Al-Ahsa, Saudi Arabia; weldeeb@kfu.edu.sa; 7 Department of Public Health, College of Veterinary Medicine, King Faisal University, P.O. Box 400, Al-Hofuf 31982, Al-Ahsa, Saudi Arabia; sdiab@kfu.edu.sa (S.M.A.-R.); falmathen@kfu.edu.sa (F.A.); salshami@kfu.edu.sa (S.A.-S.); salsultan@kfu.edu.sa (S.I.A.-S.); aalfaify@kfu.edu.sa (A.A.)

**Keywords:** *P. aeruginosa*, fish, poultry, human, AMR, *bla*_SHV_ sequencing, One Health

## Abstract

*Pseudomonas aeruginosa* is an opportunistic pathogen of public health concern. This study aimed to investigate the prevalence of *P. aeruginosa*, some virulence factors, and antimicrobial resistance patterns and highlight the potential pathways of horizontal *bla*_SHV_-resistant gene transfer from diverse sources. A total of 220 samples were collected from fish (n = 90), water (n = 30), poultry (n = 50), and humans (n = 50). All samples were isolated, confirmed by the Vitek 2 system, and tested against antimicrobial agents. Some virulence and resistance genes were examined by PCR and sequenced for the *bla*_SHV_-resistant gene from four selected isolates from each source. SPSS v26, with chi-squared tests and Pearson correlations (*p* < 0.05), was implemented for statistical investigation. *P. aeruginosa* was isolated at 33.3%, 20%, 14%, and 24% from fish, water, poultry, and humans, respectively. Using the diffusion disk method, extensively drug-resistant (XDR) and multidrug-resistant (MDR) strains were detected. All strains harbored the *opr*L and *tox*A genes, while the *las*B gene was present in 40% of fish samples but not present in human samples. All strains lacked the *exo*S gene. The *tet*A, *sul*1, *bla*_SHV_, and *bla*_TEM_ resistance genes were detected at different percentages. The *bla*_SHV_ genes from fish and water isolates were closely related to each other and showed similarity to those of the human isolates. The poultry isolates formed a separate phylogenetic lineage. The emergence of XDR and MDR *P. aeruginosa* highlights a possible public health threat. Based on the gene similarity between fish and water isolates, our results suggest that these isolates have a common origin. The similarity between the human isolates and environmental isolates (fish and water) raises concerns about possible transmission to humans.

## 1. Introduction

*Pseudomonas aeruginosa* (*P. aeruginosa*) is an opportunistic bacterium that poses a significant concern in both human and veterinary medicine due to its occurrence in several environments, including aquatic life, poultry, and water sources. In fish farming, it produces septicemia and ulceration in fish, leading to serious complications and financial losses [1]. In poultry, it can cause respiratory illnesses, septicemia, and reduced productivity, particularly in intensive farms. Polluted water is a critical source and dissemination vector for this durable pathogen [2]. In humans, *P. aeruginosa* is the primary cause of nosocomial infection and is frequently resilient to numerous medications, making treatment difficult [3]. Under the One Health approach, its importance is highlighted by its existence in the human, animal, and environmental sectors. Its ecology and patterns of transmission must be understood in order to implement integrated control procedures [4].

Fish constitute a rich source of good-quality protein and nutritional elements such as omega-3 fatty acids, selenium, vitamin D, and iodine [5]. The consumption of fish has recently seen a 3.2% annual increase in comparison to meat products, particularly in developing countries [6]. There are over 200 species in the genus *Pseudomonas*, such as *P. aeruginosa*, *P. anguilliseptica*, *P. lundensis*, *P. putida*, *P. fragi*, and *P. fluorescens* [7]. Both freshwater and marine water have an abundance of these bacteria [6]. In the poultry industry, *P. aeruginosa* is a major pathogen that primarily affects impaired hens. It can cause serious infections such septicemia, yolk sac infections, and respiratory diseases, and it is commonly isolated from hatcheries, water supply systems, litter, and poultry enclosure air [8]. The pathogen’s capacity to create biofilms increases its resistance and survival, making antimicrobial treatment and disinfection difficult. Infections can result in increased mortality, slow development rates, and financial losses in the production of broilers [9].

Numerous virulence factors are produced by *P. aeruginosa*, such as exotoxins (exotoxins S, T, U, and A), pigments (pyomelanin, pyoverdine, and pyocyanine), proteases (which cause tissue destruction), flagella, lectins, siderophores, and different secretion systems (the Type III “injectasome” in particular) [10]. The *toxA* gene encodes the extracellular enzyme exotoxin A (ETA), which prevents host cells from synthesizing proteins [11]. The type III secretion system’s (T3SS) effector exotoxins—*ExoT*, *ExoS*, *ExoY*, and *ExoU*—are believed to be a major contributor to P. aeruginosa virulence [12]. *LasB* elastase breaks down the structural proteins of cells, including elastin, collagen, and non-collagen proteins [13]. *P. aeruginosa*’s outer membrane proteins (*OprI* and *OprL*) are essential for the bacterium’s interactions with its environment and its natural resistance to antibiotics; their presence has been associated with efflux transport systems that affect cell permeability [14]. Because these proteins are specific to this organism, they could be a reliable indicator for the rapid detection of *P. aeruginosa* in clinical samples [15].

Multidrug resistance (MDR) in bacteria has emerged as one of the 21st century’s most formidable problems because of the rising incidence of illnesses that are difficult to cure and the dearth of appropriate therapeutic options [12]. Due to *P. aeruginosa*’s innate resistance to several antibiotic classes and its capacity to develop resistance to almost all potent antimicrobial agents, MDR strains can arise [16]. Among the most prevalent genes for antibiotic resistance, *bla*_SHV_ aids bacteria such as *P. aeruginosa* in their resistance to β-lactam drugs. This gene has been detected in humans, fish, poultry, and water, among other sources. According to new research on poultry, *P. aeruginosa* from broiler farms carries the *bla*_SHV_ gene, frequently in combination with other resistance genes [17]. *bla*_SHV_-positive bacteria are also found in farm and natural water, most likely as a result of pollution and animal husbandry waste. Laboratory tests have revealed a substantial frequency of *bla*_SHV_ in humans, especially in hospital-associated infections [18].

A straightforward, integrated framework is used in the One Health strategy to emphasize the connections between aquatic, animal, and human health. To understand and lessen the risks of disease transmission between species, this point of view is crucial. Recent studies have pointed out the value of such a strategy in combating resistant bacteria and newly developing transmissible diseases. Uddin et al. (2021) [12] suggested the need for continuous detection and control strategies to combat multidrug-resistant *P. aeruginosa*. Additionally, ref. [4] focused on the necessity of a One Health framework to establish the prevalence of zoonotic threats. By adopting this strategy, this study aimed to assess *P. aeruginosa*’s prevalence, virulence factors, and resistance patterns and highlight the potential pathways involved in the horizontal gene transfer and environmental dissemination of the *bla*_SHV_ gene among *P. aeruginosa* isolates from diverse sources.

## 2. Materials and Methods

### 2.1. Animal Ethics

Fish and poultry handling, human sample collection, and other experimental methods were approved by Suez Canal University’s Animal Ethics Review Committee, Egypt, with ethics number SCU-VET-AREC-R-2025018.

### 2.2. Sampling

Samples were collected from different types of sources in the Ismailia Governorate of Egypt for the isolation of *P. aeruginosa*. From three freshwater fish farms, 90 moribund fish samples (30 Oreochromis niloticus, 30 Tilapia zillii, and 30 Clarias gariepinus) were gathered. Each fish’s gills, liver, spleen, and kidneys were removed aseptically and placed in aerated plastic bags in accordance with Wamala et al. [19]. Thirty water samples were collected from the three corresponding freshwater fish farms. According to [20], water samples were handled and stored in an icebox. Lung, liver, and spleen samples were collected from 50 broiler chicks suffering from respiratory manifestations. The samples were pooled and handled as one. Fifty swabs were collected from human sputum, wounds, and urine at Suez Canal University Hospital in the Ismailia Governorate. Suez Canal University’s Faculty of Veterinary Medicine’s Bacteriology Laboratory received all samples for bacteriological examination.

### 2.3. Isolation, Vitek 2 Identification, and PCR Confirmation of Recovered Isolates

Samples were directly streaked onto cetrimide agar (Himedia, Maharashtra, India) and MacConkey’s agar (Oxoid, Cheshire, UK) and then incubated aerobically for 24 h at 37 °C. Yellowish green fluorescent pigment production is commonly associated with Pseudomonads [21]. Recovered colonies were purified to determine their biochemical and phenotypic characteristics. In short, Gram’s stain was used for the morphological identification of each isolate, and the Vitek 2 system (bioMérieux, Marcy-l’Étoile, France) was used for biochemical identification. Additionally, the detected isolates were confirmed using a species-specific set of primers that targeted the *P. aeruginosa 16SrRNA* gene (PaF: 5′-GGGGGATCTTCGGACCTCA-3′; PaR: 5′TCCTTAGAGTGCCCACCCG-3′), as outlined by Spilker et al. [22].

### 2.4. Antimicrobial Susceptibility Testing

The obtained isolates were subjected to antimicrobial susceptibility testing utilizing 11 antimicrobial agents (Oxoid, UK) from seven antibiotic classes: the tetracycline class (tetracycline (TE, 30 µg), oxytetracycline (OT, 30 µg)); the aminoglycoside class (amikacin (AK, 30 µg), tobramycin (TOB, 10 µg)); the cephalosporin III class (cefotaxime (CTX, 30 µg), ceftriaxone (CRO, 30 µg)); the β-lactam–β-lactamase inhibitor combination class (amoxicillin clavulanic acid (AMC, 30 µg), ampicillin/sulbactam (SAM, 20 µg)); the folate pathway inhibitor class (sulfamethoxazole/trimethoprim (SXT, 25 µg)); the fluoroquinolone class (ciprofloxacin (CIP, 5 µg)); and the polymyxin class (colistin (CT, 10 µg)). The test was carried out on Muller–Hinton agar plates (Oxoid, UK), which were incubated at 37 °C for 24 h. The test findings were interpreted in light of the CLSI [23].

### 2.5. Molecular Typing of Isolated P. aeruginosa Strains’ Virulence and Antimicrobial Resistance Genes

A silica-based membrane QIAamp DNA Mini Kit (catalog no. 51304, Hilden, Germany) was used to extract the DNA of purified strains in accordance with the manufacturer’s instructions. Briefly, QIAGEN protease was pipetted into a microcentrifuge tube; then, the sample was added; then, buffer AL was added, and the sample was mixed, incubated, and centrifuged; ethanol (96%) was added and it was mixed and then centrifuged again. Afterwards, the mixture was applied to the QIAamp mini spin column and centrifugated. The QIAamp mini spin column was placed in a clean 2 mL collection tube, and the tube containing the filtrate was discarded. The QIAamp mini spin column was then opened, buffer AW1 was added, and it was centrifuged. The QIAamp mini spin column was next placed in a clean 2 mL collection tube, and the tube containing the filtrate was discarded. Then, the QIAamp mini spin column was opened, buffer AW2 was added, and it was centrifuged. The QIAamp mini spin column was then placed in a new 2 mL collection tube and centrifuged. Following this, the QIAamp mini spin column was placed in a clean microcentrifuge; then, buffer AE was added, and it was incubated and then centrifuged. Genomic DNA templates were quantified using Nanodrop (Nanodrop 1000, Thermo Scientific, London, UK) and afterwards kept at −20 °C until they were required for PCR. Virulence genes (*opr*L, *tox*A, *las*B, and *exo*S) and antimicrobial resistance genes (*tet*A, *sul*1, *bla*_SHV,_ and *bla*_TEM_) were detected in the *P. aeruginosa* strains. The retrieved bacteria’s resistance to commercially available antibiotics was confirmed by the selection of antimicrobial-resistant genes. All primers were provided by the Metabion Company in Germany; Table 1 lists the oligonucleotide sequences and PCR settings for each primer. In a T100 gradient thermocycler (Biometra, Jena, Germany), PCR reactions (25 μL) were amplified using the EmeraldAmp GT PCR Master Mix (Code No. RR310A, Takara, Japan). The positive control was a virulent reference strain of *P. aeruginosa* that was generously supplied by the Animal Health Research Institute in Dokki, Cairo, Egypt. This strain was multidrug-resistant to tetracycline, sulfamethoxazole/trimethoprim, ceftriaxone, and cefotaxime. Meanwhile, a reaction without template DNA functioned as a negative control. After screening the products using 1.5% (*w*/*v*) agarose gel electrophoresis (AppliChem GmbH, Darmstadt, Germany), they were photographed.

### 2.6. Sequence Analysis of bla_SHV_-Resistant Gene

The *bla*_SHV_ gene was sequenced for four *P. aeruginosa* strains that encoded the *bla_SHV_*-resistant gene (one strain from fish, water, poultry, and humans). On an Applied Biosystems 3130 automated DNA sequencer (Applied Biosystems, Thermo Fisher Scientific, Carlsbad, CA, USA), a purified PCR product was sequenced in both the forward and reverse directions using a ready reaction Bigdye Terminator V3.1 cycle sequencing kit (Perkin-Elmer/Applied Biosystems, Foster City, CA, USA), with Cat. No. 4336817. According to Altschul et al. [24], a Basic Local Alignment Search Tool (BLAST) analysis was first conducted to determine sequence identity to GenBank accessions. Mesquite v.3.40, equipped with Clustal v.2.1, was utilized to align and trim the acquired sequences with the existing sequences from GenBank. The jModelTest v.2.1.6 implementation of the Akaike information criterion was used to select the evolution models (TIM3 + F). Using the maximum likelihood approach, a phylogenetic tree was constructed using IQ-Tree v.1.6.10.

### 2.7. Statistical Analysis

SPSS version 26 (IBM Corp, Armonk, NY, USA) was used to analyze the data. The chi-squared test was used to analyze the categorical data, including the differences in the prevalence of *P. aeruginosa* isolates from different sources, and to assess the differences in the antimicrobial resistance patterns of the recovered isolates from various sources and the existence of different tested genes among the investigated isolates of different origins. The correlations between the tested variables were estimated by Pearson’s correlations (*r*). Pearson’s correlations (*r*) were estimated and visualized using the Hmisc package [25] in the R software, version 4.3.3 (https://www.r-project.org/, accessed on 10 July 2025). Before correlation analyses, variables were tested for normality using a Q-Q plot. The *opr*L, *tox*A, and *exo*S gene variables were excluded from the analyses as they were identical among all isolates under study. The *p*-values were considered statistically significant if they were less than 0.05. All graphs were generated by the R software, version 4.3.3 [26], using ggplot [27], the Hmisc package [25], and the pheatmap package [28] to generate a radar chart, heatmap, hierarchical clustering heatmap, box plot, stacked bar plot, and correlation plots.

## 3. Results

### 3.1. Phenotypic Characteristics of Isolated P. aeruginosa from Different Sources

All recovered isolates were arranged microscopically in double or short chains of Gram-negative bacilli. Typically, *P. aeruginosa* isolates obtained on cetrimide agar at 37 °C for 24 h had a yellowish green fluorescent pigment and exhibited irregular, large colonies with a fruity odor. On MacConkey’s agar, the bacteria proliferated and appeared as fat, smooth, non-lactose-fermenting colonies with regular edges and an alligator skin appearance from the top view. The Vitek 2 results revealed that the probability of *P. aeruginosa* identification reached 99%. Additionally, all isolates showed positive results in the PCR amplification of the species-specific *16S rRNA* gene.

### 3.2. Prevalence of P. aeruginosa from Different Sources

**Figure 1:**
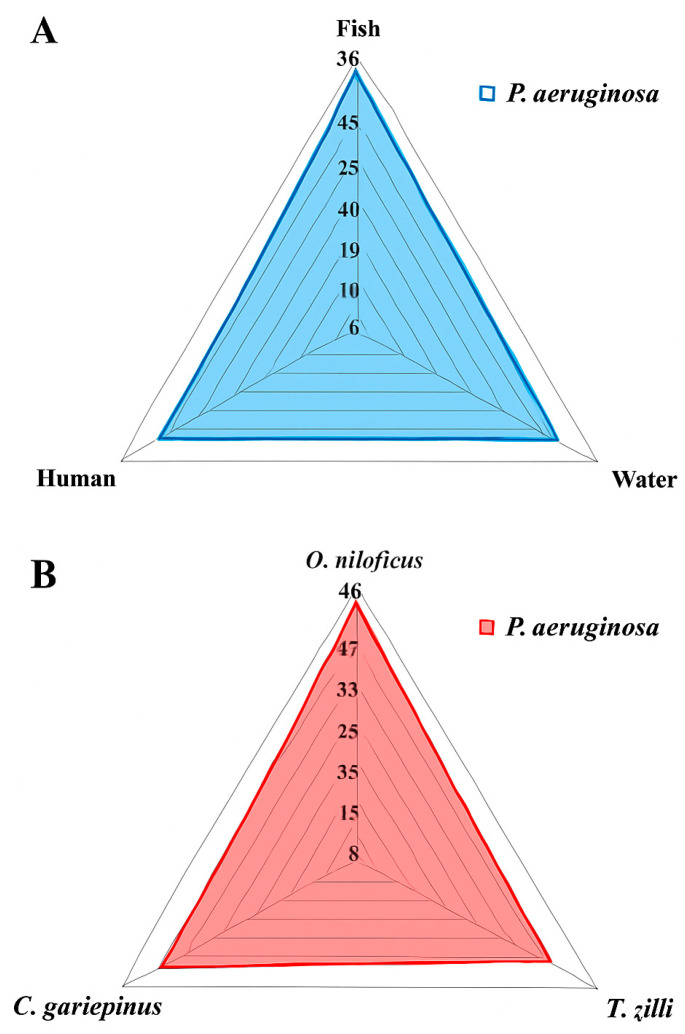
Prevalence of *P. aeruginosa* isolated from different locations (**A**) and fish species (**B**).

**Figure 2:**
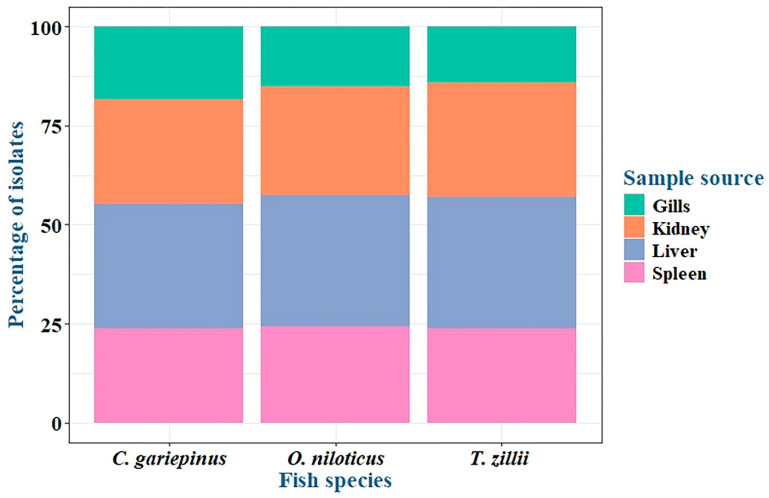
*P. aeruginosa* prevalence in organs of infected fish species.

Among the examined fish, the overall prevalence of *P. aeruginosa* was 33.3% (30/90) (Table 2 and Figure 1). The highest prevalence was recorded in Clarias gariepinus (12/30, 40%), followed by Oreochromis niloticus (11/30, 36.6%) and then Tilapia zillii (7/30, 23.3%) (Table 3 and Figure 1). There was no statistically significant difference in the prevalence of *P. aeruginosa* among various fish species (*p* > 0.05). Concerning *P. aeruginosa*’s prevalence in various infected organs, the most common infected organ was the liver, followed by the kidney, spleen, and gills (Table 3 and Figure 2). Statistically, the prevalence of *P. aeruginosa* among different organs in the examined fish species showed no significant difference (*p* > 0.05). The overall *P. aeruginosa* prevalence in water, poultry, and humans was 20%, 14%, and 24%, respectively (Table 2 and Figure 1). The *P. aeruginosa* prevalence across different human samples was 50% (6/12) in wounds, 33.3% (4/12) in sputum, and 16.7% (2/12) in urine. There was no significant difference in the dissemination of *P. aeruginosa* among the examined samples from different sources (*p* = 0.064).

### 3.3. P. aeruginosa Antimicrobial Susceptibility Testing

**Figure 3:**
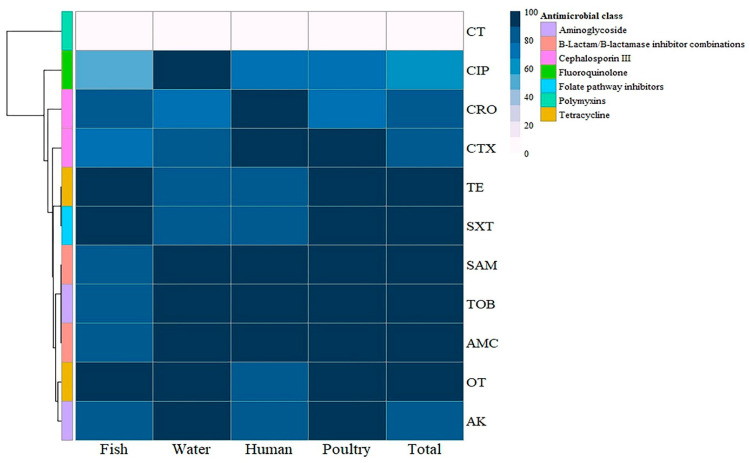
Hierarchical clustering heatmap showing the antibiogram of the isolated *P. aeruginosa* from different sources. The color code on the right of the heatmap refers to the resistance percentage to the tested antimicrobial agent. TE: Tetracycline, OT: Oxytetracycline, AK: Amikacin, TOB: Tobramycin, CTX: Cefotaxime, CRO: Ceftriaxone, AMC: Amoxycillin/Clavulanic Acid, SAM: Ampicillin/Sulbactam, SXT: Sulfamethoxazole/Trimethoprim, CIP: Ciprofloxacin, CT: Colistin.

The antimicrobial susceptibility of the retrieved *P. aeruginosa* isolates from fish consisted of remarkable resistance to tetracycline (100%), sulfamethoxazole/trimethoprim (100%), oxytetracycline (93%), amikacin (83%), tobramycin (83%), amoxicillin clavulanic acid (83%), ampicillin/sulbactam (83%), ceftriaxone (80%), and cefotaxime (73%). Moreover, 100% of isolates were sensitive to colistin. *P. aeruginosa* isolates from water showed 100% resistance to oxytetracycline, amikacin, tobramycin, amoxicillin clavulanic acid, ampicillin/sulbactam, and ciprofloxacin and 83% resistance to tetracycline, cefotaxime, and sulfamethoxazole/trimethoprim. Additionally, they showed 67% resistance to ceftriaxone but were 100% sensitive to colistin. *P. aeruginosa* isolates from humans showed 100% resistance to tobramycin, cefotaxime, ceftriaxone, amoxicillin, clavulanic acid, and ampicillin/sulbactam; 83% resistance to tetracycline, oxytetracycline, amikacin, and sulfamethoxazole/trimethoprim; and 67% resistance to ciprofloxacin. However, all human *P. aeruginosa* isolates were sensitive to colistin, reaching 100%. *P. aeruginosa* isolates from poultry showed 100% resistance to tetracycline, oxytetracycline, ampicillin/sulbactam, amoxicillin, clavulanic acid, cefotaxime, sulfamethoxazole/trimethoprim, amikacin, and tobramycin, while 71.4% of isolates were sensitive to colistin, as shown in Table 4 and Figure 3. Statistical analysis revealed no significant variations (*p* > 0.05) in the antimicrobial resistance patterns of the tested *P. aeruginosa* isolates from various sources to all tested antimicrobials.

### 3.4. Virulence and Antimicrobial Resistance Genes of P. aeruginosa Strains

**Figure 4:**
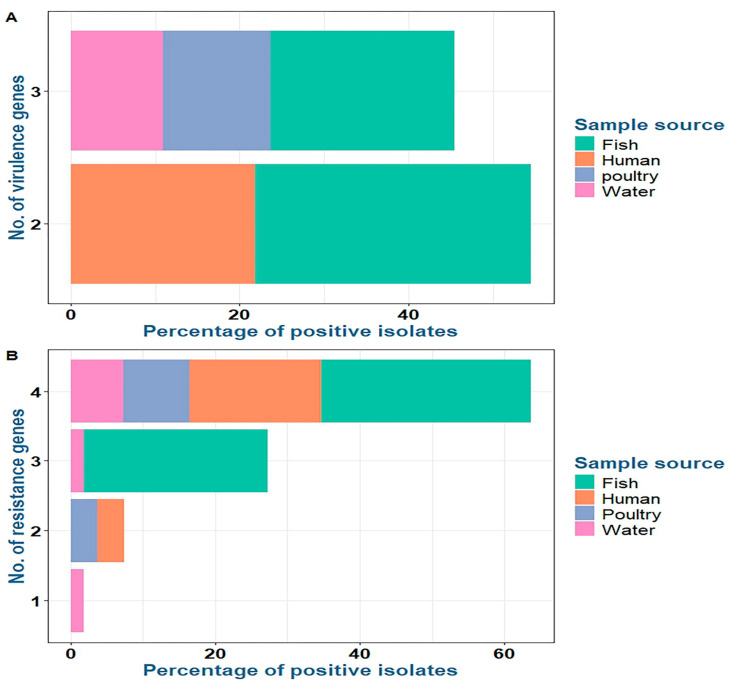
Distribution of virulence (**A**) and resistance (**B**) genes among *P. aeruginosa* isolates from various sources.

**Figure 5:**
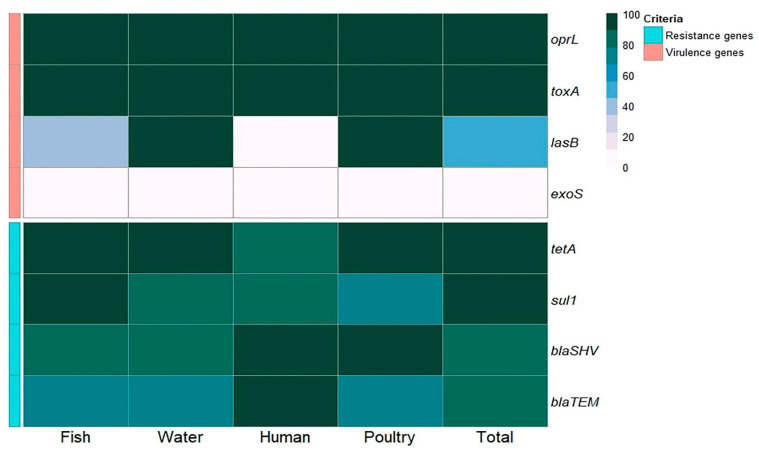
Frequency of antibiotic resistance and virulence genes in *P. aeruginosa* isolates obtained from various sources.

Concerning virulence genes, the recovered *P. aeruginosa* strains isolated from fish commonly harbored the virulence genes *opr*L (100%) and *tox*A (100%), followed by *las*B (40%), while the *exo*S gene was not detected (0%). *P. aeruginosa* strains isolated from water and poultry commonly harbored the virulence genes *opr*L (100%), *tox*A (100%), and *las*B (100%), while the *exo*S gene was not detected (0%). *P. aeruginosa* strains isolated from humans commonly harbored the virulence genes *opr*L (100%) and *tox*A (100%), while the *las*B and *exo*S genes were not detected (0%) (Table 5 and Figure 4 and Figure 5).

Concerning antimicrobial resistance genes, the recovered *P. aeruginosa* strains isolated from fish commonly harbored *tet*A (tetracycline resistance) (100%), *sul*1 (sulfonamide resistance) (100%), and *bla*_SHV_ (β-lactam resistance) (80%), followed by *bla*_TEM_ (β-lactam resistance) (73.3%). *P. aeruginosa* strains isolated from water harbored *tet*A (100%), *sul*1 (83.3%), and *bla*_SHV_ (83.3%), followed by *bla*_TEM_ (66.7%). *P. aeruginosa* strains isolated from poultry harbored *tet*A, *bla*_SHV_, *sul*1, and *bla*_TEM_ at 100%, 100%, 71.4%, and 71.4%, respectively. *P. aeruginosa* strains isolated from humans harbored *bla*_SHV_, *bla*_TEM_, *tet*A, and *sul*1 at 100%, 100%, 83.3%, and 83.3%, respectively (Table 5 and Figure 4 and Figure 5). There was a statistically significant difference in the prevalence of the *las*B and *sul*1 genes among *P. aeruginosa* isolates from different sources (*p* < 0.001). There was no statistically significant difference in the distributions of *tet*A, *bla*_SHV_, and *bla*_TEM_ resistance genes among *P. aeruginosa* isolates from different sources (*p* > 0.05). There were statistically significant differences in the distributions of two and three resistance genes (*p* < 0.001) and two and three resistance genes (*p* = 0.021, 0.004) among *P. aeruginosa* isolated from fish, water, poultry, and human samples. There were no statistically significant differences in the distributions of one and four virulence genes among *P. aeruginosa* isolated from fish, water, poultry, and human samples (*p* = 0.109 and 0.322, respectively) (Table 5).

### 3.5. Patterns of Multidrug Resistance in Recovered P. aeruginosa Strains

The study’s findings indicated that 53.3% (16/30) of the retrieved *P. aeruginosa* isolates obtained from fish were XDR to six antimicrobial classes (tetracyclines: TE and OT; folate pathway inhibitors: SXT; aminoglycosides: AK and TOB; β-lactam–β-lactamase inhibitor combinations: SAM and AMC; cephalosporin III: CTX and CRO; and fluoroquinolone: CIP) and harbored the *tet*A, *sul*1, *bla*_TEM,_ and *bla*_SHV_ resistance genes. Moreover, 16.7% (5/30) of the isolated *P. aeruginosa* strains were MDR to five different classes (tetracyclines: TE and OT; folate pathway inhibitors: SXT; β-lactam–β-lactamase inhibitor combinations: SAM and AMC; aminoglycosides: AK and TOB; and cephalosporin III: CRO) and harbored the *tet*A, *sul*1, and *bla*_SHV_ resistance genes. In addition, 13.3% (4/30) of the isolated *P. aeruginosa* strains were MDR to five different classes (tetracyclines: TE and OT; folate pathway inhibitors: SXT; aminoglycosides: AK and TOB; β-lactam–β-lactamase inhibitor combinations: SAM and AMC; and cephalosporin III: CTX) and harbored the *tet*A, *sul*1, and *bla*_TEM_ resistance genes. Moreover, 10% (3/30) of the isolated *P. aeruginosa* strains were MDR to three classes (tetracyclines: TE and OT; folate pathway inhibitors: SXT; and cephalosporin III: CTX) and harbored the *tet*A, *sul*1, and *bla*_SHV_ resistance genes. In addition, 6.7% (2/30) of the isolated *P. aeruginosa* strains were MDR to three classes (tetracyclines: TE; folate pathway inhibitors: SXT; and cephalosporin III: CTX) and harbored the *tet*A, *sul*1, and *bla*_TEM_ resistance genes (Table 6).

Concerning *P. aeruginosa* isolates obtained from water, 66.6% (4/6) were XDR to six antimicrobial classes (tetracyclines: TE and OT; β-lactam–β-lactamase inhibitor combinations: SAM and AMC; cephalosporin III: CTX and CRO; folate pathway inhibitors: SXT; fluoroquinolone: CIP; and aminoglycosides: TOB and AK) and harbored the *tet*A, *sul*1, *bla*_SHV_, and *bla*_TEM_ resistance genes. In addition, 16.7% (1/6) were XDR to six antimicrobial classes (tetracyclines: TE and OT; β-lactam–β-lactamase inhibitor combinations: SAM and AMC; cephalosporin III: CTX; folate pathway inhibitors: SXT; fluoroquinolone: CIP; and aminoglycosides: TOB and AK) and harbored the *tet*A, *sul*1, and *bla*_SHV_ resistance genes. Moreover, 16.7% (1/6) were MDR to four antimicrobial classes (tetracyclines: OT; β-lactam–β-lactamase inhibitor combinations: SAM and AMC; fluoroquinolone: CIP; and aminoglycosides: TOB and AK) and harbored the *tet*A and *bla*_SHV_ resistance genes (Table 6).

Concerning *P. aeruginosa* isolates obtained from poultry, 71.4% (5/7) were XDR to six antimicrobial classes (tetracyclines: TE and OT; β-lactam–β-lactamase inhibitor combinations: SAM and AMC; cephalosporin III: CTX and CRO; folate pathway inhibitors: SXT; fluoroquinolone: CIP; and aminoglycosides: TOB and AK) and harbored the *tet*A, *sul*1, *bla*_SHV_, and *bla*_TEM_ resistance genes. In addition, 28.6% (2/7) were MDR to five antimicrobial classes (tetracyclines: TE and OT; β-lactam–β-lactamase inhibitor combinations: SAM and AMC; cephalosporin III: CTX; folate pathway inhibitors: SXT; and aminoglycosides: TOB and AK) and harbored the *tet*A and *bla*_SHV_ resistance genes (Table 6).

Regarding *P. aeruginosa* isolates obtained from humans, 66.7% (8/12) were XDR to six antimicrobial classes (aminoglycosides: TOB and AK; tetracyclines: TE and OT; cephalosporin III: CTX and CRO; β-lactam–β-lactamase inhibitor combinations: SAM and AMC; fluoroquinolone: CIP; and folate pathway inhibitors: SXT) and harbored the *tet*A, *sul*1, *bla*_SHV_, and *bla*_TEM_ resistance genes. In addition, 16.7% (2/12) were MDR to five antimicrobial classes (tetracyclines: TE and OT; cephalosporin III: CTX and CRO; β-lactam–β-lactamase inhibitor combinations: SAM and AMC; aminoglycosides: TOB; and folate pathway inhibitors: SXT) and harbored the *tet*A, *sul*1, *bla*_SHV_, and *bla*_TEM_ resistance genes. Moreover, 16.7% (2/12) were MDR to three antimicrobial classes (aminoglycosides: TOB and AK; cephalosporin III: CTX and CRO; and β-lactam–β-lactamase inhibitor combinations: SAM and AMC) and harbored the *tet*A, *sul*1, *bla*_SHV_, and *bla*_TEM_ resistance genes (Table 6). The conducted analysis showed that, throughout all examined sources, XDR isolates were more prevalent than MDR isolates. Sixteen fish isolates, five water isolates, five poultry isolates, and eight human isolates were found to be XDR, while 14, 1, 2, and 4 isolates from the same sources were found to be MDR. The higher incidence of XDR over MDR poses challenges in providing proper treatment and emphasizes the urgent need for integrated One Health programs and the improved oversight of antibiotics to prevent subsequent transmission.

**Figure 6:**
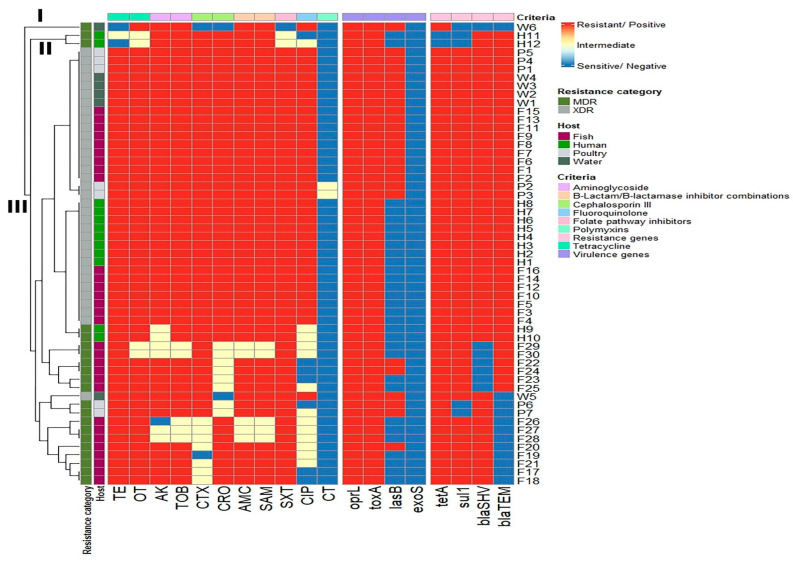
Hierarchical clustering heatmap depicting the overall distribution of the *P. aeruginosa* isolates depending on their phenotypic antimicrobial resistance patterns, resistance genes, and virulence genes. Different sample sources, resistance categories, and antimicrobial classes are color-coded on the right side of the heatmap. TE: Tetracycline, OT: Oxytetracycline, AK: Amikacin, TOB: Tobramycin, CTX: Cefotaxime, CRO: Ceftriaxone, AMC: Amoxycillin/Clavulanic Acid, SAM: Ampicillin/Sulbactam, SXT: Sulfamethoxazole/Trimethoprim, CIP: Ciprofloxacin, CT: Colistin.

The clustering patterns of *P. aeruginosa* isolates based on the phenotypic antimicrobial resistance genes and virulence genes displayed medium diversity and polyclonality. Of the 55 examined isolates, only 12 isolates belonged to various lineages. Moreover, three main branches (I, II, and III) and eight clusters were observed in our results, and close relatedness was determined between *P. aeruginosa* isolates from human and fish samples (Figure 6).

**Figure 7:**
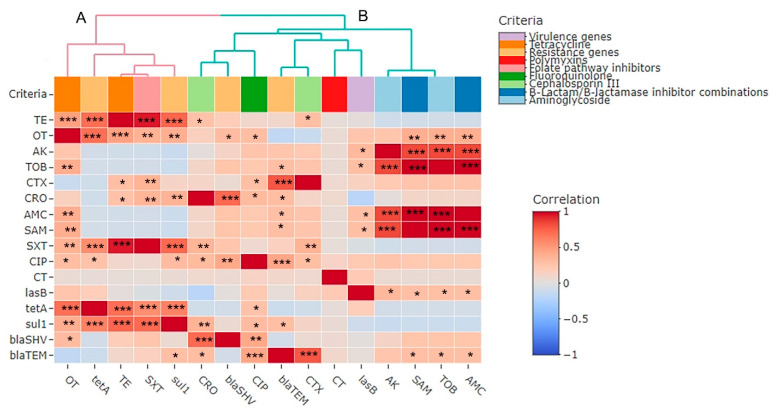
Pairwise correlations (*r*) between phenotypic antimicrobial resistance genes and virulence genes of *P. aeruginosa* isolates exhibited with two branches (**A**,**B**). The scale on the right of the figure refers to the correlation coefficient (*r*). Stars refer to a significant correlation: * *p* < 0.05, ** *p* < 0.01, *** *p* < 0.001. Variables that are identical among all strains are excluded and thus not shown in this figure. TE: Tetracycline, OT: Oxytetracycline, AK: Amikacin, TOB: Tobramycin, CTX: Cefotaxime, CRO: Ceftriaxone, AMC: Amoxycillin/Clavulanic Acid, SAM: Ampicillin/Sulbactam, SXT: Sulfamethoxazole/Trimethoprim, CIP: Ciprofloxacin, CT: Colistin.

The relatedness of the investigated phenotypic antimicrobial resistance genes and virulence genes exhibited low diversity, with two branches (A and B) and one cluster. The SAM, AMC, and TOB antimicrobials were clustered together. Additionally, we identified a highly positive, significant correlation between the TE and OT phenotypes and the *tet*A (r = 0.69 each, *p* < 0.001) and *sul*1 (r = 0.73 and 0.4, respectively, *p* < 0.01) genes, as well as a significant (*p* < 0.0001) positive correlation between the *tet*A and *sul1* genes (r = 0.61). We determined a significant (*p* < 0.05) positive correlation between the *las*B gene and the SAM, AMC, TOB, and AK phenotypes (r = 0.29, 0.29, 0.29, and 0.33, respectively). Moreover, the *bla*_SHV_ gene correlated significantly and positively with the OT (r = 0.31, *p*-value < 0.05), CIP (r = 0.43, *p*-value < 0.01), and CRO (r = 0.72, *p*-value < 0.0001) phenotypes, and there was a significant positive correlation between the *bla*_TEM_ gene and the CTX, CIP (r = 0.78 and 0.44, respectively, *p*-value < 0.0001), CRO (r = 0.3, *p* < 0.05), TOB, AMC, and SAM phenotypes, as well as the *sul*1 gene (r = 0.29 each, *p*-value < 0.05) (Figure 7).

**Figure 8:**
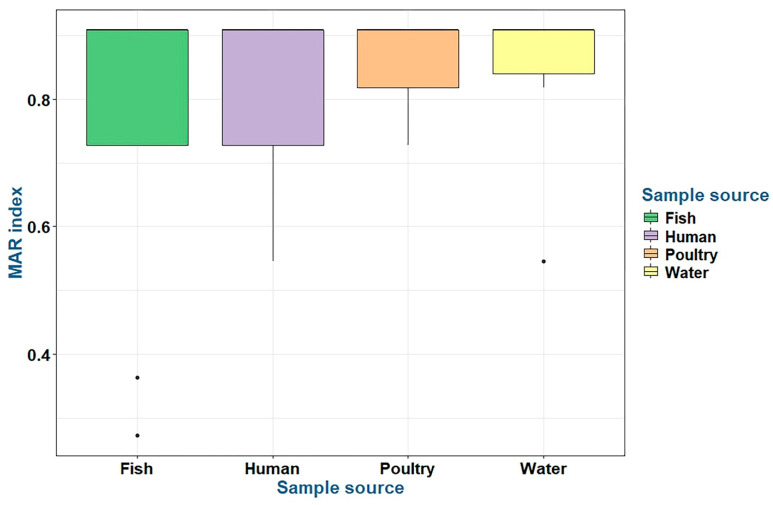
The multiple antibiotic resistance (MAR) indices of the tested *P. aeruginosa* isolates belonging to various sources.

*P. aeruginosa* strains recovered from fish, water, poultry, and humans had MAR index values ranging from 0.27 to 0.91 (Table 7 and Figure 8). There were no statistically significant differences in the prevalence of MAR indices and resistance to antimicrobial classes (*p* > 0.05) among *P. aeruginosa* isolates obtained from fish, water, poultry, and human samples (Table 7).

### 3.6. Sequence Analysis of bla_SHV_ Gene in P. aeruginosa Strains from Fish, Water, and Humans

**Figure 9:**
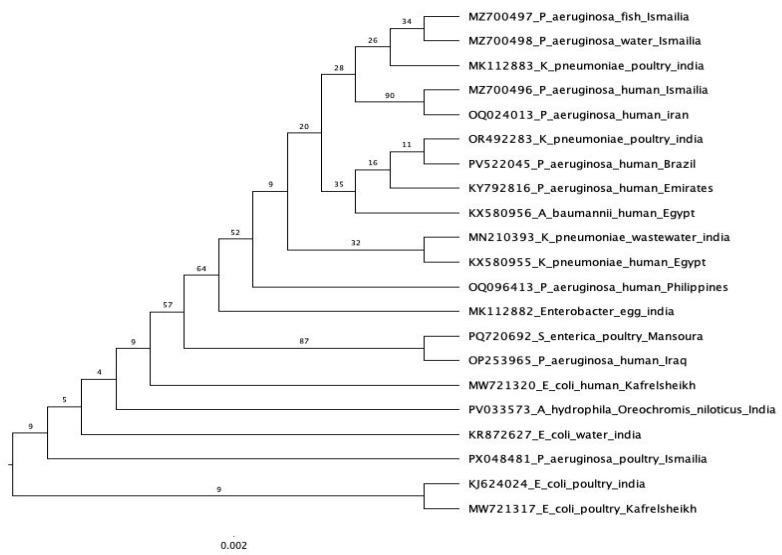
Phylogenetic analysis of the *bla*_SHV_ gene sequence from different sources. All other sequences are derived from the NCBI GenBank database.

Four *P. aeruginosa* isolates—originating from fish, water, poultry, and human sources—were selected for phylogenetic analysis based on their phenotypic resistance to β-lactam antibiotics and the presence of the *bla*_SHV_ gene, as confirmed by molecular screening. Sequencing of the *bla*_SHV_ gene was performed for each isolate. The sequences obtained from the fish and water isolates were submitted to GenBank under the accession numbers MZ700497 and MZ700498 (Figure 9), respectively. Comparative sequence analysis revealed a high degree of similarity between these two environmental isolates, suggesting a close genetic relationship or potentially a common origin.

The *bla*_SHV_ gene sequence from the human isolate, submitted under GenBank accession number MZ700496 (Figure 9), exhibited high similarity to *bla*_SHV_-1 sequences previously reported from clinical *P. aeruginosa* strains in Iran. Interestingly, this human-derived sequence also showed greater similarity to the sequences from the fish and water isolates in the Ismailia Governorate than to the poultry-derived isolate.

The *bla*_SHV_ gene sequence from the poultry isolate was submitted under GenBank accession number PX048481 (Figure 9). Phylogenetic analysis placed the poultry-derived sequence in a distinct clade, separate from the other sources analyzed in this study. Notably, this sequence exhibited greater similarity to a *bla*_SHV_ gene from *E. coli* isolated from water in India, rather than to any of the local isolates examined.

## 4. Discussion

*Pseudomonas aeruginosa* is recognized as a significant pathogen responsible for ulcerative infections in fish populations. These infections have elevated mortality rates, caused substantial economic losses, and reduced efficiency in fish farming [16], in addition to having zoonotic implications [29]. In the current study, *P. aeruginosa*’s overall prevalence among the analyzed fish was 33.3%. The highest incidence was seen in Clarias gariepinus (40%), followed by Oreochromis niloticus (36.6%) and then Tilapia zillii (23.3%). The liver was the most significantly affected organ. The results aligned with [18,30], which found that the *P. aeruginosa* prevalence in fish was 31.57% and 31.5%, respectively. Meanwhile, ref. [31] found a higher incidence of *P. aeruginosa* in Clarias gariepinus than in Oreochromis niloticus. Moreover, ref. [1] regularly isolated *P. aeruginosa* from liver samples.

The studies [7,19,31] revealed a decrease in the *P. aeruginosa* incidence rates in fish of 12%, 4.2%, and 13.8%, respectively. Geographical dispersion, environmental factors, host vulnerability, and the sample collection season may all contribute to variations in prevalence. *P. aeruginosa* was discovered in 20% of the water samples, which differed from [32], where the authors isolated *P. aeruginosa* from various water sources, such as lakes, ponds, and rivers, with a percentage of 61.5%. The status of the water in which the fish were hunted is considered an indicator of the bacterial load; hence, fish could acquire harmful microorganisms from their natural aquatic habitats [33].

It is notable that *P. aeruginosa* was isolated from poultry at a percentage of 14%. In large-scale chicken farms, where even a small percentage of *P. aeruginosa* might affect a large number of birds, this percentage may appear insignificant, yet it represents a notable concern. Our results were nearly identical to those in [34], which stated that the isolation rate of *P. aeruginosa* in broiler chickens was 18% in Beni-Suef and Fayoum, Egypt. In contrast, ref. [35] recorded a high isolation rate of *P. aeruginosa* from broiler chickens in Fayoum, Egypt, reaching 77.5%. These findings are within the ranges reported in other countries. This percentage is significant from a clinical and financial standpoint because *P. aeruginosa* is not a typical component of the poultry flora and is frequently linked to illness outbreaks. *P. aeruginosa* infections can result in low weight gain, high chick mortality, and a greater need for antibiotics. Furthermore, it may serve as a covert source of infection or antibiotic resistance in birds that appear to be in good health [36]. As a result, even a 14% rate would necessitate regular screening, improved sanitation, and conscientious antibiotic use in chicken farms.

The results revealed a high isolation rate of *P. aeruginosa* from the pus of infected wounds and burns in human samples (50%), followed by sputum (33.3%) and then urine samples (16.7%). This finding is almost in line with the study [37] regarding the isolation rate of *P. aeruginosa* from pus (50%), followed by sputum (25%) and then urine samples (16.67%). In contrast, ref. [38] found that the organism was responsible for 16% of nosocomial pneumonia cases, 11% of hospital-acquired urinary tract infections, and 8% of surgical wound infections. Differences across studies may arise from differing hospital conditions and sample sizes.

The long-term use of antimicrobial drugs to treat *Pseudomonas* infections leads to the emergence of multidrug-resistant strains in aquatic environments via R-plasmid transfer [39]. According to [40], *P. aeruginosa* is resistant to several antibiotics, including aminoglycosides, quinolones, and β-lactams. Regarding antimicrobial sensitivity tests, all isolates recovered from different sources did not show resistance to colistin, a result confirmed in [18], which found *P. aeruginosa* to be 100% sensitive to colistin. In contrast, ref. [41] described the 100% resistance of *P. aeruginosa* isolates to colistin. Furthermore, all isolates showed resistance to other tested antimicrobial agents, with different percentages. These findings were consistent with the data in [42], where the authors determined the high resistance of *P. aeruginosa* to ampicillin/sulbactam and amoxicillin/clavulanic acid (68%).

The current data highlight XDR *P. aeruginosa* resistance to six antimicrobial classes, including the *tet*A, *sul*1, *bla*_TEM_, and *bla*_SHV_ resistance genes. These findings are congruent with those in [18,43]. The indiscriminate use of antibiotics and evolving antibiotic resistance genes may lead to the emergence of MDR strains [44]. The MAR value in this investigation was above 0.2, echoing the results of [43]. The survival ability of *P. aeruginosa* strains in a polluted environment is indicated by an MAR value of more than 0.2, and the high rate of deterioration in the samples under investigation may have been caused by the presence of several antibiotic residues [45]. The evolution of numerous and widespread drug-resistant *P. aeruginosa* strains with public health implications is highlighted in this study; as a result, preventing food poisoning requires proper handling, storage, and transportation procedures, as well as antimicrobial drug surveillance systems [46]. Furthermore, MDR *P. aeruginosa* strains were found in three or five antimicrobial classes and had the *tet*A, *sul*1, *bla*_TEM_, or *bla*_SHV_ resistance genes. These findings align with [47], where the authors detected a high rate of resistance for tetracyclines and low resistance for colistin, and 24.3% of isolates were MDR. Meanwhile, ref. [48] revealed 97.5% resistance to sulfamethoxazole/trimethoprim and [49] detected colistin sensitivity amounting to 85%, amikacin resistance of 64%, and ceftriaxone and cefotaxime resistance of 56.52%, with 32.8% of *P. aeruginosa* isolates from humans. In contrast, in [49,50], the authors discovered the lowest resistance to amikacin. The difference in bacterial resistance prevalence rates among studies may arise from many factors, including the type of clinical specimen investigated, community hygiene, and antimicrobial agent exposure [48]. *P. aeruginosa* is broadly resistant to numerous medications and becomes progressively resistant to most existing antibiotics, making it a major concern in hospital settings all over the world [51].

According to the current study’s PCR results, every tested strain was positive for the *opr*L and *tox*A genes, which is in line with [35]. *P. aeruginosa*’s outer membrane proteins, known as L-lipoproteins, give the bacterium resistance to antiseptics and other antimicrobial agents. Because they are limited to *Pseudomonad* species, they may be a valuable target for identifying and assessing the virulence of *Pseudomonads* in clinical specimens [52]. The *tox*A gene on the virulent *P. aeruginosa* chromosome encodes exotoxin A, an extracellular component of the bacterium. It functions similarly to the diphtheria toxin in that it prevents the host cell from producing proteins [53]. The *las*B gene was absent in human isolates, while it was found in 40%, 100%, and 100% of fish, poultry, and water isolates, respectively. Moreover, ref. [54] detected *las*B at 41.2%. *Las*B hydrolyzes various host proteins, destroying host tissues and the immune response and promoting inflammation [55]. In this research, the *exo*S gene was absent in all strains, and this is almost aligned with [55], where the authors detected a minor prevalence of the *exo*S gene (5%). Antimicrobial resistance genes were distributed in different percentages in all isolates, which is in line with [18,56]. These resistant and highly virulent bacteria can colonize new environments and cause disease more efficiently [57].

The phylogenetic relationships observed in this study highlight potential pathways of horizontal gene transfer and environmental dissemination of the *bla*_SHV_ gene among *P. aeruginosa* isolates from diverse sources. The close genetic similarity between the fish and water isolates suggests a shared environmental reservoir or direct contamination linkage, likely facilitated by aquatic ecosystems. The similarity between the human isolates and environmental isolates (fish and water) raises concerns about possible transmission from environmental sources to humans, especially in regions with inadequate wastewater management. In contrast, the poultry isolates formed a separate phylogenetic lineage, indicating a distinct origin or the acquisition of the *bla*_SHV_ gene, potentially influenced by agricultural antimicrobial usage. The similarity between the poultry isolates and an *E. coli* strain from India may reflect the international dissemination of resistance genes through the food chain or animal trade. These findings underscore the importance of integrated One Health surveillance approaches to monitor and control the spread of antibiotic resistance genes across the environmental, animal, and human health sectors. Our results follow [58], where the authors stated that bacteria are widespread in the aquatic habitat and can cause significant mortality in wild and cultivated fish because of illnesses caused by numerous bacterial species. Most bacterial pathogens are a part of the natural microflora prevalent in aquatic environments; however, they can also act as opportunistic or secondary infections. Many patients acquire infections linked to *P. aeruginosa*, posing a public health concern [18]. Nowadays, *Pseudomonas* species cause foodborne diseases that humans can acquire by eating contaminated food and prepared goods, handling contaminated seafood [59], or by eating raw fish and its byproducts [60]. The potential for antimicrobial-resistant gene expression in *P. aeruginosa* to circulate among individuals, poultry, fish, and water supplies constitutes one of the most notable public health implications highlighted by our results. Tracking and combating these infections on a global scale is vital, since the high incidence and resistance patterns found in our isolates indicate that these sources may serve as repositories for resilient strains. Our results support the urgent need for comprehensive antimicrobial oversight along with consolidated One Health policies to prevent the spread of resistance and maintain human and animal health.

## 5. Conclusions

Our study revealed the concerning prevalence of XDR and MDR *P. aeruginosa* isolates from fish, water, poultry, and humans in Egypt. Remarkably, all strains harbored the *opr*L and *tox*A virulence genes. The *tet*A, *sul*1, *bla*_SHV_, and *bla*_TEM_ resistance genes were also detected at high prevalences in all isolates. The close genetic similarity between the fish and water isolates suggests a shared environmental reservoir or direct contamination linkage, likely facilitated by aquatic ecosystems. The similarity between the human isolates and environmental isolates (fish and water) raises concerns about possible transmission to humans. These findings underscore the urgent need for stringent control measures to mitigate the spread of MDR and XDR *P. aeruginosa* and to address the misuse of antimicrobials in fish farming, in line with the One Health perspective. The relatively high detection rate of the *bla*_SHV_ gene can be interpreted in light of local epidemiological and ecological conditions, where environmental reservoirs, antimicrobial selection pressure, and horizontal gene transfer may facilitate the dissemination of resistance determinants across bacterial species. It is therefore expected that detection rates may vary between different geographic regions.

## Figures and Tables

**Table 1:** PCR conditions for testing of *P. aeruginosa* encoding genes.

Gene	Primary Denaturation	Secondary Denaturation	Annealing	Extension	Final Extension	Amplified Product (bp)	Reference
***P. aeruginosa*** ***16S rRNA***	95 °C 5 min	94 °C 30 s	52 °C 30 s	72 °C 50 s	72 °C 10 min	956	[22]
***opr*L**		94 °C 30 s	55 °C 40 s	72 °C 45 s	72 °C 10 min	504	[18]
***tox*A**		94 °C 30 s	55 °C 40 s	72 °C 40 s	72 °C 7 min	396	
***las*B**		94 °C 30 s	54 °C 40 s	72 °C 1 min	72 °C 12 min	1220	
***exo*S**		94 °C 30 s	55 °C 30 s	72 °C 30 s	72 °C 7 min	118	
***bla*_TEM_**		94 °C 30 s	54 °C 40 s	72 °C 45 s	72 °C 7 min	516	
***bla*_SHV_**		94 °C 30 s	54 °C 40 s	72 °C 40 s	72 °C 10 min	392	
***sul*1**		94 °C 30 s	60 °C 40 s	72 °C 45 s	72 °C 10 min	433	
***tet*A**		94 °C 30 s	50 °C 40 s	72 °C 45 s	72 °C 10 min	576	

**Table 2:** Prevalence of *P. aeruginosa* isolated from different sources.

Source of Sample	No. of Samples	Isolated *P. aeruginosa*	
		Number	%
**Fish**	90	30	33.3
**Water**	30	6	20
**Poultry**	50	7	14
**Humans**	50	12	24
**Total**	220	55	25
***p*-value**		0.064	

**Table 3:** Prevalence of *P. aeruginosa* isolated from different fish organs.

Fish Species (n = 30 Each)	No. of Positive Fish (%)	Total No. of Isolates	No. and Percentage of *P. aeruginosa* in Various Organs (n = 30 Each)				*p*-Value
			Liver	Kidney	Spleen	Gills	
***Oreochromis niloticus***	11 (36.7)	33	11 (33.3)	9 (27.3)	8 (24.2)	5 (15.2)	0.413
***Tilapia zillii***	7 (23.3)	21	7 (33.3)	6 (28.6)	5 (23.8)	3 (14.3)	0.634
***Clarias gariepinus***	12 (40)	38	12 (31.6)	10 (26.3)	9 (23.7)	7 (18.4)	0.597
***p*-value**	0.458		0.458	0.581	0.554	0.439	
**Total (n = 90)**	30 (33.3)	92	30 (32.6)	25 (27.2)	22 (23.9)	15 (16.3)	0.077

**Table 4:** The antimicrobial resistance patterns of the isolated *P. aeruginosa* from different sources.

Antimicrobial Class	Antimicrobial Agent	No. of *P. aeruginosa* Isolates (%)				*p*-Value	Total no. of *P. aeruginosa* Isolates (%) (n = 55)
		Fish (n = 30)	Water (n = 6)	Humans (n = 12)	Poultry (n = 7)		
Tetracycline	TE	30 (100)	5 (83.3)	10 (83.3)	7 (100)	0.092	52 (94.5)
	OT	28 (93.3)	6 (100)	10 (83.3)	7 (100)	0.477	51 (92.7)
Aminoglycoside	AK	25 (83.3)	6 (100)	10 (83.3)	7 (100)	0.591	48 (87.3)
	TOB	25 (83.3)	6 (100)	12 (100)	7 (100)	0.269	50 (90.9)
Cephalosporin III	CTX	22 (73.3)	5 (83.3)	12 (100)	7 (100)	0.103	46 (87.3)
	CRO	24 (80)	4 (66.7)	12 (100)	5 (71.4)	0.263	45 (81.8)
β-Lactam/β-lactamase inhibitor combinations	AMC	25 (83.3)	6 (100)	12 (100)	7 (100)	0.269	50 (90.9)
	SAM	25 (83.3)	6 (100)	12 (100)	7 (100)	0.269	50 (90.9)
Folate pathway inhibitors	SXT	30 (100)	5 (83.3)	10 (83.3)	7 (100)	0.090	52 (94.5)
Fluoroquinolone	CIP	16 (53.3)	6 (100)	8 (66.7)	5 (71.4)	0.172	35 (63.6)
Polymyxins	CT	0	0	0	0	NA	0
*p*-value		<0.001	<0.001	<0.001	<0.001		

TE: Tetracycline, OT: Oxytetracycline, AK: Amikacin, TOB: Tobramycin, CTX: Cefotaxime, CRO: Ceftriaxone, AMC: Amoxycillin/Clavulanic Acid, SAM: Ampicillin/Sulbactam, SXT: Sulfamethoxazole/Trimethoprim, CIP: Ciprofloxacin, CT: Colistin, NA: not applicable.

**Table 5:** Prevalence of virulence and antimicrobial resistance genes among *P. aeruginosa* strains from different sources by PCR.

Type of Gene		Number and Percentage of *P. aeruginosa*				*p*-Value	Total Number and Percentage of Isolates (n = 55)
		Fish (n = 30)	Water (n = 6)	Poultry (n = 7)	Humans (n = 12)		
Virulence genes	*opr*L	30 (100)	6 (100)	7 (100)	12 (100)	NA	55 (100)
	*tox*A	30 (100)	6 (100)	7 (100)	12 (100)	NA	55 (100)
	*las*B	12 (40)	6 (100)	7 (100)	0	<0.001 ***	25 (45.5)
	*exo*S	0	0	0	0	NA	0
***p*-value**		<0.001	<0.001	<0.001	<0.001		
Antimicrobial resistance genes	*tet*A	30 (100)	6 (100)	7 (100)	10 (83.3)	0.069	53 (96.4)
	*sul*1	30 (100)	5 (83.3)	5 (71.4)	10 (83.3)	0.036 *	50 (90.9)
	*bla*_SHV_	24 (80)	5 (83.3)	7 (100)	12 (100)	0.188	48 (87.3)
	*bla*_TEM_	22 (73.3)	4 (66.7)	5 (71.4)	12 (100)	0.218	43 (78.2)
***p*-value**		0.001	0.878	0.28	0.307		

NA: not applicable, * *p* < 0.05, *** *p* < 0.001.

**Table 6:** Multidrug resistance patterns and antimicrobial resistance gene distribution among *P. aeruginosa* strains (n = 30 for fish, n = 6 for water, n = 7 for poultry, and n = 12 for humans).

Origin	No. of Strains	%	Type of Resistance	Multidrug Resistance Profile	Antimicrobial Resistance Genes
Fish	16	53.3	XDR	- Six classes: (TE and OT), (SXT), (AK and TOB), (SAM and AMC), (CTX and CRO), (CIP)	*tet*A, *sul*1, *bla*_TEM_, and *bla*_SHV_
	5	16.7	MDR	- Five classes: (TE and OT), (SXT), (CRO), (SAM and AMC), (AK and TOB)	*tet*A, *sul*1, and *bla*_SHV_
	4	13.3	MDR	- Five classes: (TE and OT), (SXT), (AK and TOB), (SAM and AMC), (CTX)	*tet*A, *sul*1, and *bla*_TEM_
	3	10	MDR	- Three classes: (TE and OT), (SXT), (CRO)	*tet*A, *sul*1, and *bla*_SHV_
	2	6.7	MDR	- Three classes: (TE), (SXT), (CTX)	*tet*A, *sul*1, and *bla*_TEM_
Water	4	66.6	XDR	- Six classes: (TE and OT), (SAM and AMC), (CTX and CRO), (SXT), (CIP), (TOB and AK)	*tet*A, *sul*1, *bla*_SHV_, and *bla*_TEM_
	1	16.7	XDR	- Six classes: (TE and OT), (SAM and AMC), (CTX), (SXT), (CIP), (TOB and AK)	*tet*A, *sul*1, and *bla*_SHV_
	1	16.7	MDR	- Four classes: (OT), (SAM and AMC), (CIP), (TOB and AK)	*tet*A and *bla*_SHV_
Poultry	5	71.4	XDR	- Six classes: (TE and OT), (SAM and AMC), (CTX and CRO), (SXT), (CIP), (TOB and AK)	*tet*A, *sul*1, *bla*_SHV_, and *bla*_TEM_
	2	28.6	MDR	- Five classes: (TE and OT), (SAM and AMC), (TOB and AK), (CTX), (SXT)	*tet*A, *bla*_SHV_
Humans	8	66.7	XDR	- Six classes: (TOB and AK), (TE and OT), (CTX and CRO), (SAM and AMC), (CIP), (SXT)	*tet*A, *sul*1, *bla*_SHV_, and *bla*_TEM_
	2	16.7	MDR	- Five classes: (TE and OT), (CTX and CRO), (SAM and AMC), (TOB), (SXT)	*tet*A, *sul*1, *bla*_SHV_, and *bla*_TEM_
	2	16.7	MDR	- Three classes: (TOB and AK), (CTX and CRO), (SAM and AMC)	*bla*_SHV_ and *bla*_TEM_

**Table 7:** Frequency of resistance to various antimicrobial agents in *P. aeruginosa* isolates belonging to various sources.

MAR Index	AMA	No. of *P. aeruginosa* Isolates (%)				*p*-Value	Total No. of *P. aeruginosa* Isolates (%) (n = 55)
		Fish (n = 30)	Water (n = 6)	Humans (n = 12)	Poultry (n = 7)		
0.27	3	2 (6.67)	0	0	0	0.758	2 (3.6)
0.36	4	3 (10)	0	0	0	0.507	3 (5.5)
0.55	6	0	1 (16.7)	2 (16.7)	0	0.092	3 (5.5)
0.73	8	9 (30)	0	2 (16.7)	2 (28.6)	0.459	13 (23.6)
0.82	9	0	1 (16.7)	0	0	0.109	1 (1.8)
0.91	10	16 (53.3)	4 (66.7)	8 (66.7)	5 (71.4)	0.763	33 (60)

AMA: antimicrobial agent. MAR: multiple antibiotic resistance.

## Data Availability

All data generated or analyzed during this study are included in this published article.

## References

[1] Abou Elez R.M.M., Zahra E.M.F., Gharieb R.M.A., Mohamed M.E.M., Samir M., Saad A.M., Merwad A.M.A. (2024). Resistance patterns, virulence determinants, and biofilm genes of multidrug-resistant *P. aeruginosa* isolated from fish and fish handlers. Sci. Rep..

[2] Marouf S., Li X., Salem H.M., Ahmed Z.S., Nader S.M., Shaalan M., Awad F.H., Zhou H., Cheang T. (2023). Molecular detection of multidrug-resistant *P. aeruginosa* of different avian sources with pathogenicity testing and in vitro evaluation of antibacterial efficacy of silver nanoparticles against multidrug-resistant *P. aeruginosa*. Poult. Sci..

[3] Reynolds D., Kollef M. (2021). The Epidemiology and Pathogenesis and Treatment of *P. aeruginosa* Infections: An Update. Drugs.

[4] World Health Organization (2024). WHO Bacterial Priority Pathogens List, 2024: Bacterial Pathogens of Public Health Importance, to Guide Research, Development, and Strategies to Prevent and Control Antimicrobial Resistance.

[5] Morshdy A.E.M., Hussein M.A., Mohamed M.A.A., Hamed E., El-Murr A.E., Darwish W.S. (2022). Tetracycline residues in tilapia and catfish tissue and the effect of different cooking methods on oxytetracycline and doxycycline residues. J. Consum. Prot. Food Saf..

[6] Ben Mhenni N., Alberghini G., Giaccone V., Truant A., Catellani P. (2023). Prevalence and antibiotic resistance phenotypes of *Pseudomonas* spp. in fresh fish fillets. Foods.

[7] Abd El Tawab A.A., Maarouf A.A., Ahmed N.M. (2016). Detection of Virulence factors of *Pseudomonas* species isolated from fresh water fish by PCR. Benha Vet. Med. J..

[8] Rizk A.M., Elsayed M.M., Abd El Tawab A.A., Elhofy F.I., Soliman E.A., Kozytska T., Brangsch H., Sprague L.D., Neubauer H., Wareth G. (2024). Phenotypic and genotypic characterization of resistance and virulence in *P. aeruginosa* isolated from poultry farms in Egypt using whole genome sequencing. Vet. Microbiol..

[9] Odoi H., Boamah V.E., Boakye Y.D., Agyare C. (2021). Prevalence and Phenotypic and Genotypic Resistance Mechanisms of Multidrug-Resistant *P. aeruginosa* Strains Isolated from Clinical, Environmental, and Poultry Litter Samples from the Ashanti Region of Ghana. J. Environ. Public Health.

[10] Behzadi P., Baráth Z., Gajdács M. (2021). It’s not easy being green: A narrative review on the microbiology, virulence and therapeutic prospects of multidrug-resistant *P. aeruginosa*. Antibiotics.

[11] Horii T., Muramatsu H., Monji A., Miyagishima D. (2005). Release of exotoxin A, peptidoglycan and endotoxin after exposure of clinical *P. aeruginosa* isolates to carbapenems in vitro. Chemotherapy.

[12] Uddin T.M., Chakraborty A.J., Khusro A., Zidan B.R.M., Mitra S., Emran T.B., Dhama K., Ripon M.K.H., Gajdács M., Sahibzada M.U.K. (2021). Antibiotic resistance in microbes: History, mechanisms, therapeutic strategies and future prospects. J. Infect. Public Health.

[13] Chadha J., Harjai K., Chhibber S. (2022). Revisiting the virulence hallmarks of *P. aeruginosa*: A chronicle through the perspective of quorum sensing. Environ. Microbiol..

[14] Sabzehali F., Rahimi H., Goudarzi H., Goudarzi M., Izad M.H.Y., Chirani A.S., Jalali S.A., Faghihloo E. (2021). Functional engineering of OprF-OprI-PopB as a chimeric immunogen and its cross-protective evaluation with GM-CSF against *P. aeruginosa*: A comprehensive immune informatics evaluation. Inform. Med. Unlocked.

[15] Zhao Y., Xu H., Wang H., Wang P., Chen S. (2024). Multidrug resistance in *P. aeruginosa*: Genetic control mechanisms and therapeutic advances. Mol Biomed.

[16] Shahrokhi G.R., Rahimi E., Shakerian A. (2022). The prevalence rate, pattern of antibiotic resistance, and frequency of virulence factors of *P. aeruginosa* strains isolated from fish in Iran. J. Food Qual..

[17] Islam R., Ferdous F.B., Hoque M.N., Asif N.A., Rana M.L., Siddique M.P., Rahman M.T. (2024). Characterization of beta-lactamase and virulence genes in *P. aeruginosa* isolated from clinical, environmental and poultry sources in Bangladesh. PLoS ONE.

[18] Algammal A.M., Mabrok M., Sivaramasamy E., Youssef F.M., Atwa M.H., El-Kholy A.W., Hetta H.F., Hozzein W.N. (2020). Emerging MDR- *P. aeruginosa* in fish commonly harbor opr L and tox A virulence genes and bla TEM, bla CTX-M, and tet A antibiotic-resistance genes. Sci. Rep..

[19] Wamala S.P., Mugimba K.K., Mutoloki S., Evensen Ø., Mdegela R., Byarugaba D.K., Sørum H. (2018). Occurrence and antibiotic susceptibility of fish bacteria isolated from *Oreochromis niloticus* (Nile tilapia) and *Clarias gariepinus* (African catfish) in Uganda. Fish. Aquat. Sci..

[20] Yisa J., Tijani J.O. (2010). Analytical studies on water quality index of river Landzu. Am. J. Appl. Sci..

[21] Lamont I.L., Martin L.W. (2003). Identification and characterization of novel pyoverdine synthesis genes in *P. aeruginosa*. Microbiology.

[22] Spilker T., Coenye T., Vandamme P., LiPuma J.J. (2004). PCR-based assay for differentiation of *P. aeruginosa* from other Pseudomonas species recovered from cystic fibrosis patients. J. Clin. Microbiol..

[23] Clinical and Laboratory Standards Institute (CLSI) (2016). Performance Standards for Antimicrobial Susceptibility Testing.

[24] Altschul S.F., Gish W., Miller W., Myers E.W., Lipman D.J. (1990). Basic local alignment search tool. J. Mol. Biol..

[25] Harrell F.E. (2003). Hmisc: Harrell Miscellaneous. CRAN Contrib. Packag..

[26] R Core Team (2024). R: A Language and Environment for Statistical Computing.

[27] Wickham H., Chang W., Henry L., Pedersen T.L., Takahashi K., Wilke C., Woo K., Yutani H., Dunnington D., van den Brand T. (2007). ggplot2: Create Elegant Data Visualisations Using the Grammar of Graphics. CRAN Contrib. Packag..

[28] Kolde R. Pheatmap: Pretty heatmaps. https://CRAN.R-project.org/package=pheatmap.

[29] Abdulhaq N., Nawaz Z., Zahoor M.A., Siddique A.B. (2020). Association of biofilm formation with multi drug resistance in clinical isolates of *P. aeruginosa*. EXCLI J..

[30] Sowmya R., Chinta S.S., Sreedevi B., Suresh Y., Vinayaka Siddhartha P., Madhava Rao T. (2024). Molecular characterization and antibiogram of *P. aeruginosa* isolated from fish sold in markets of Tirupati, India: Molecular characterization and antibiogram of *P. aeruginosa*. Fish. Technol..

[31] Ali H., Awad A., Maarouf A. (2023). Molecular Detection of some Virulence Factors of *P. aeruginosa* Isolated from Freshwater Fishes at Qalubiya Governorate, Egypt. Benha Vet. Med. J..

[32] Nasreen M., Sarker A., Malek M., Ansaruzzaman M., Rahman M. (2015). Prevalence and resistance pattern of *P. aeruginosa* isolated from surface water. Adv. Microbiol..

[33] Alawy A.E., El-Tras W.F., El Raiy H.R., Khater D.F. (2015). Impact of industrial wastewater on water and fish quality of Nile River in Kafr El-Zayat, Egypt. Benha Vet. Med. J..

[34] Hassan W.H., Ibrahim A.M.K., Shany S.A.S., Salam H.S.H. (2020). Virulence and resistance determinants in *P. aeruginosa* isolated from pericarditis in diseased broiler chickens in Egypt. J. Adv. Vet. Anim. Res..

[35] Morsi A.I., Abdallah M., Rafequ A., Elkhayat M. (2024). Incidence and virulence gene profiling of *P. aeruginosa* in broiler chickens from Fayoum Governorate, Egypt. Benha Vet. Med. J..

[36] Abd El-Ghany W.A. (2021). *Pseudomonas aeruginosa* infection of avian origin: Zoonosis and one health implications. Vet. World.

[37] Ismail M., El Lamei M. (2017). Studies on *P. septicemia* in some tilapia in Ismailia. Suez Canal Vet. Med. J..

[38] Ikeno T., Fukuda K., Ogawa M., Honda M., Tanabe T., Taniguchi H. (2007). Small and rough colony *P. aeruginosa* with elevated biofilm formation ability isolated in hospitalized patients. Microbiol. Immunol..

[39] Abd-El-Maogoud H.A.-E.-N., Edris A.B.M., Mahmoud A.H., Maky M.A. (2021). Occurrence and characterization of *Pseudomonas* species isolated from fish marketed in Sohag Governorate, Egypt. SVU-Int. J. Vet. Sci..

[40] Hancock R.E., Brinkman F.S. (2002). Function of *Pseudomonas* porins in uptake and efflux. Annu. Rev. Microbiol..

[41] Akhi M.T., Ghotaslou R., Beheshtirouy S., Asgharzadeh M., Pirzadeh T., Asghari B., Alizadeh N., Ostadgavahi A.T., Somesaraei V.S., Memar M.Y. (2015). Antibiotic susceptibility pattern of aerobic and anaerobic bacteria isolated from surgical site infection of hospitalized patients. Jundishapur J. Microbiol..

[42] Abd El-Baky R.M., Masoud S.M., Mohamed D.S., Waly N.G., Shafik E.A., Mohareb D.A., Elkady A., Elbadr M.M., Hetta H.F. (2020). Prevalence and some possible mechanisms of colistin resistance among multidrug-resistant and extensively drug-resistant *P. aeruginosa*. Infect. Drug Resist..

[43] Shalmashi H., Farajnia S., Sadeghi M., Tanoumand A., Veissi K., Hamishekar H., Gotaslou R. (2022). Detection of ESBLs types blaCTX-M, blaSHV and blaTEM resistance genes among clinical isolates of *P. aeruginosa*. Gene Rep..

[44] Farhan S.M., Ibrahim R.A., Mahran K.M., Hetta H.F., Abd El-Baky R.M. (2019). Antimicrobial resistance pattern and molecular genetic distribution of metallo-β-lactamases producing *P. aeruginosa* isolated from hospitals in Minia, Egypt. Infect. Drug Resist..

[45] Talukder A., Rahman M.M., Chowdhury M.M.H., Mobashshera T.A., Islam N.N. (2021). Plasmid profiling of multiple antibiotic-resistant *P. aeruginosa* isolated from soil of the industrial area in Chittagong, Bangladesh. Beni-Suef Univ. J. Basic Appl. Sci..

[46] Tiamiyu A., Soladoye M., Adegboyega T., Adetona M. (2015). Occurrence and antibiotic sensitivity of bacterial strains isolated from Nile tilapia, Oreochromis niloticus obtained in Ibadan, Southwest Nigeria. J. Biosci. Med..

[47] Bahador N., Shoja S., Faridi F., Dozandeh-Mobarrez B., Qeshmi F.I., Javadpour S., Mokhtary S. (2019). Molecular detection of virulence factors and biofilm formation in *P. aeruginosa* obtained from different clinical specimens in Bandar Abbas. Iran. J. Microbiol..

[48] Hasan S.A., Najati A.M., Abass K.S. (2020). Prevalence and antibiotic resistance of *P. aeruginosa* isolated from clinical samples in Kirkuk City, Iraq. Eurasian J. Biosci..

[49] Pokharel K., Dawadi B.R., Bhatt C.P., Gupte S. (2019). Prevalence of *P. aeruginosa* and its Antibiotic Sensitivity Pattern. J. Nepal Health Res. Counc..

[50] Deodurg P.M., Doddamani P.K., Rana S., Mir B.A. (2014). Prevalence and Antimicrobial Susceptibility Pattern of *P. aeruginosa* in a Tertiary Care Hospital. Res. J. Pharm. Technol..

[51] Buhl M., Peter S., Willmann M. (2015). Prevalence and risk factors associated with colonization and infection of extensively drug-resistant *P. aeruginosa*: A systematic review. Expert Rev. Anti-Infect. Ther..

[52] Remans K., Vercammen K., Bodilis J., Cornelis P. (2010). Genome-wide analysis and literature-based survey of lipoproteins in *P. aeruginosa*. Microbiology.

[53] Aljebory I.S. (2018). PCR detection of some virulence genes of *P. aeruginosa* in Kirkuk city, Iraq. J. Pharm. Sci. Res..

[54] Hassuna N.A., Mandour S.A., Mohamed E.S. (2020). Virulence constitution of multi-drug-resistant *P. aeruginosa* in upper Egypt. Infect. Drug Resist..

[55] Gaviard C., Cosette P., Jouenne T., Hardouin J. (2019). LasB and CbpD virulence factors of *P. aeruginosa* carry multiple post-translational modifications on their lysine residues. J. Proteome Res..

[56] Hosu M.C., Vasaikar S.D., Okuthe G.E., Apalata T. (2021). Detection of extended spectrum beta-lactamase genes in *P. aeruginosa* isolated from patients in rural Eastern Cape Province, South Africa. Sci. Rep..

[57] Beceiro A., Tomás M., Bou G. (2013). Antimicrobial resistance and virulence: A successful or deleterious association in the bacterial world?. Clin. Microbiol. Rev..

[58] Zaheen Z., War A.F., Ali S., Yatoo A.M., Ali M.N., Ahmad S.B., Rehman M.U., Paray B.A. (2022). Common bacterial infections affecting freshwater fish fauna and impact of pollution and water quality characteristics on bacterial pathogenicity. Bacterial fish diseases.

[59] Gram L., Huss H.H. (2000). Fresh and processed fish and shellfish. The Microbiological Safety and Quality of Food.

[60] Novotny L., Dvorska L., Lorencova A., Beran V., Pavlik I. (2004). Fish: A potential source of bacterial pathogens for human beings. Veterinární Medicína.

