# Correction: Wahdan et al. One Health Monitoring of Resistant *Pseudomonas aeruginosa* in Aquatic, Poultry, and Human Sources: Virulence Traits and *bla*_SHV_ Gene Tracking. *Pathogens* 2025, *14*, 983

**DOI:** 10.3390/pathogens15050481

**Published:** 2026-04-30

**Authors:** Ali Wahdan, Mahmoud Ezzat, Amal Emam, Walaa A. Husseiny, Mohamed Abou El-Atta, Ehab M. Abd-Allah, Ahmed M. A. Meligy, Sherief M. Abdel-Raheem, Faisal Almathen, Salah Al-Shami, Saad I. Al-Sultan, Ahmed Alfifi, Wael El-Deeb, Marwa E. Abo Hashem

**Affiliations:** 1 Department of Bacteriology, Immunology, and Mycology, Faculty of Veterinary Medicine, Suez Canal University, Ismailia 41522, Egypt; mezzat05@yahoo.com (M.E.); marwashassan@vet.suez.edu.eg (M.E.A.H.); 2 Veterinary Medicine Directorate, Ministry of Agriculture, Ismailia 41522, Egypt; amalemam222@gmail.com; 3 Department of Animal Wealth Development, Faculty of Veterinary Medicine, Suez Canal University, Ismailia 41522, Egypt; walaa.ali@vet.suez.edu.eg; 4 Central Lab for Aquaculture Research, Abbassa 44662, Egypt; drmohamed.abouelatta@yahoo.com; 5 Veterinary Hospital, Faculty of Veterinary Medicine, Zagazig University, Zagazig 44511, Egypt; zzarad@yahoo.com; 6 Department of Clinical Science, Central Lab, College of Veterinary Medicine, King Faisal University, P.O. Box 400, Al-Hofuf 31982, Al-Ahsa, Saudi Arabia; weldeeb@kfu.edu.sa; 7 Department of Public Health, College of Veterinary Medicine, King Faisal University, P.O. Box 400, Al-Hofuf 31982, Al-Ahsa, Saudi Arabia; sdiab@kfu.edu.sa (S.M.A.-R.); falmathen@kfu.edu.sa (F.A.); salshami@kfu.edu.sa (S.A.-S.); salsultan@kfu.edu.sa (S.I.A.-S.); aalfaify@kfu.edu.sa (A.A.)

This notice provides additional clarification to the original publication [1]. The authors would like to add a clarification regarding the high prevalence of *bla*_SHV_ findings in the Conclusion section. Additionally, the Institutional Review Board Statement was incomplete.

A correction has been made to the Conclusion section, and the following statement was added: “The relatively high detection rate of the *bla*_SHV_ gene can be interpreted in light of local epidemiological and ecological conditions, where environmental reservoirs, antimicrobial selection pressure, and horizontal gene transfer may facilitate the dissemination of resistance determinants across bacterial species. It is therefore expected that detection rates may vary between different geographic regions.”

The authors propose the following clarification regarding the Institutional Review Board Statement:

“The research protocol for this study was initially approved in 2017 by the Animal Ethics Review Committee, Faculty of Veterinary Medicine, Suez Canal University, Egypt (Approval Code No. 2017011), prior to the commencement of sample collection from different sources and laboratory analyses. As this study represents part of a long-term research program, the ethical approval was formally renewed in 2025 (Ref. No. SCU-VET-AREC-R-2025018). According to the institutional policies and national regulations, the Scientific Research Ethics Committee of Suez Canal University reviewed and approved the protocol.”

The authors state that the scientific conclusions are unaffected. This correction was approved by the Academic Editor. The original publication has also been updated.
